# Holding the Inflammatory System in Check: TLRs and NLRs

**DOI:** 10.1155/2016/8156816

**Published:** 2016-08-25

**Authors:** Eda K. Holl, Irving C. Allen, Jennifer Martinez

**Affiliations:** ^1^Department of Surgery, Duke University, Durham, NC 27710, USA; ^2^Department of Biomedical Sciences and Pathobiology, Virginia-Maryland College of Veterinary Medicine, Virginia Tech, Blacksburg, VA 24061, USA; ^3^Immunity, Inflammation and Disease Laboratory, National Institute of Environmental Health Sciences, Research Triangle Park, NC 27709, USA

Aberrant inflammation has been linked to the development of a diverse spectrum of human diseases. We now appreciate that Toll-like receptor (TLR) and Nod-like receptor (NLR) signaling pathways play an important role in the onset and/or severity of these pathologies, and targeting of these receptors has shown promise in preclinical studies of infectious diseases, autoimmune disorders, tissue injury, and cancer. Recently, clinical trials have been initiated to establish the efficacy of compounds targeting TLR and NLR pathways to improve disease outcomes. Despite advancements in the field, major challenges remain in potential ways to target these receptors at various stages of disease pathogenesis and develop effective therapeutics.

The articles contained in this special issue include reviews, translational studies, and basic science findings that are focused on characterizing the contribution and molecular mechanisms associated with TLR and NLR signaling in diverse disease processes. Together, these studies serve to illustrate the essential role that these pattern recognition receptors play in maintaining immune system homeostasis in both human disease pathobiology and preclinical animal models.

In particular, this special issue emphasizes the role of TLRs and NLRs in modulating signaling pathways associated with inflammatory disease processes and cytokine production. For example, in the article entitled “Effect of Toll-Like Receptor 4 on Synovial Injury of Temporomandibular Joint in Rats Caused by Occlusal Interference” by J. Kong et al., the authors show that activation of TLR4 participates in the initiation and development of synovial membrane inflammation by regulating the expression of inflammatory mediators like IL-1*β*. This inflammation reaction and increased IL-1*β* could be restrained by treatment with a TLR4 signaling inhibitor. In addition to TLR signaling and synovial injury, the importance of NLR family members is also explored in a series of articles evaluating their contribution either following traumatic brain injury (TBI) or in the context of parasite infection. There are currently several therapeutics targeting NLR family members under development to treat or attenuate brain injury following trauma. In this special issue, T. Brickler and colleagues examine the role of the NLRP1 inflammasome in TBI. In their article entitled “Nonessential Role for the NLRP1 Inflammasome Complex in a Murine Model of Traumatic Brain Injury” the authors conclude that the NLRP1 inflammasome has only a minor role in TBI using a moderate controlled cortical impact injury model. While some cytokine expression differences were observed in* Nlrp1* ^−/−^ mice, these animals showed no significant difference in motor recovery, cell death, or contusion volume, as compared to wild-type animals. Beyond the canonical NLRP1 inflammasome, the contribution of the recently described noncanonical NLR inflammasome is also explored in the context of parasite infection and toxoplasmosis. In the article entitled “Caspase-11 Modulates Inflammation and Attenuates* Toxoplasma gondii* Pathogenesis” by S. L. Coutermarsh-Ott et al., the authors explore the contribution of this unique inflammasome signaling pathway. Here, the authors show that caspase-11 functions to protect the host by enhancing systemic inflammation during the early phase of* Toxoplasma gondii* infection and subsequently minimizes disease pathogenesis and brain cyst development during later stages of toxoplasmosis.

In addition to the preclinical animal studies described above, a trio of articles are also included in this special issue evaluating TLR and NLR regulation of inflammation as potential therapeutic targets in human disease. In the research article entitled “Maternal Vitamin D Level Is Associated with Viral Toll-Like Receptor Triggered IL-10 Response but Not the Risk of Infectious Diseases in Infancy” by S.-L. Liao et al., the authors investigate the role of maternal and cord blood vitamin D levels in TLR-mediated innate immune responses and its effect on infectious disease outcomes. The study concludes that maternal vitamin D, but not cord vitamin D, is inversely correlated with viral TLR-triggered IL-10 responses, yet it does not impact the risk of infectious disease in infancy. In addition to this research article, the review article by Z. Dong and colleagues, entitled “Holding the Inflammatory System in Check: TLRs and Their Targeted Therapy in Asthma,” focuses on describing the role of TLRs and NLRs in asthma. While not often considered major components of allergic airway inflammation, the authors present a compelling overview of ways in which these receptors contribute to disease exacerbation and potential therapeutic avenues for targeting asthma in human patients. This article suggests that combination therapies of well-timed corticosteroids and TLR agonists may represent a more effective way to control inflammation in asthmatic patients. Finally, G. Lopez-Castejon and M. J. Edelmann present a highly mechanistic perspective on the role of deubiquitinases in TLR and NLR signaling. In the review article entitled “Deubiquitinases: Novel Therapeutic Targets in Immune Surveillance?” the authors review the role of deubiquitinases in the NF-*κ*B pathway and inflammasome activation, two intrinsically related events triggered by the activation of the membrane TLRs as well as the cytosolic NLRs. The article also discusses the advances and challenges of using deubiquitinases as therapeutic targets during pathological inflammation.

With the ever-expanding realization of the role that inflammation plays in disease pathogenesis, we hope that this special issue will be of interest to the scientific community involved in studying TLRs and NLRs in the context of aberrant inflammation. We further hope that these articles emphasize the diverse function of these pattern recognition receptors in maintaining immune system homeostasis in a variety of human diseases.

*Eda K. Holl* *Eda K. Holl* *Irving C. Allen* *Irving C. Allen* *Jennifer Martinez* *Jennifer Martinez*

